# Endosomal traffic disorders: a driving force behind neurodegenerative diseases

**DOI:** 10.1186/s40035-024-00460-7

**Published:** 2024-12-24

**Authors:** Jianru Dong, Weiwei Tong, Mingyan Liu, Mengyu Liu, Jinyue Liu, Xin Jin, Ju Chen, Huachao Jia, Menglin Gao, Minjie Wei, Ying Duan, Xin Zhong

**Affiliations:** 1 School of Pharmacy, China Medical University, Shenyang, 110122 China; 2 Weifang Hospital of Traditional Chinese Medicine, Weifang, 261000 China; 3 Department of Laboratory Medicine, Shengjing Hospital of China Medical University, Shenyang, 110069 China; 4 Liaoning Medical Diagnosis and Treatment Center, Shenyang, 110167 China; 5 Liaoning Maternal and Child Health Hospital, Shenyang, 110005 China

**Keywords:** Endosomal traffic disorders, Glycolipid metabolism, Autophagy, Immunity, Neurodegenerative disease, Pathological protein aggregation, Neuroinflammation, Dysfunctional synapses and neural network, DNA and RNA defects, Therapeutic drugs

## Abstract

Endosomes are crucial sites for intracellular material sorting and transportation. Endosomal transport is a critical process involved in the selective uptake, processing, and intracellular transport of substances. The equilibrium between endocytosis and circulation mediated by the endosome-centered transport pathway plays a significant role in cell homeostasis, signal transduction, and immune response. In recent years, there have been hints linking endosomal transport abnormalities to neurodegenerative diseases, including Alzheimer's disease. Nonetheless, the related mechanisms remain unclear. Here, we provide an overview of endosomal-centered transport pathways and highlight potential physiological processes regulated by these pathways, with a particular focus on the correlation of endosomal trafficking disorders with common pathological features of neurodegenerative diseases. Additionally, we summarize potential therapeutic agents targeting endosomal trafficking for the treatment of neurodegenerative diseases.

**Graphical abstract:**

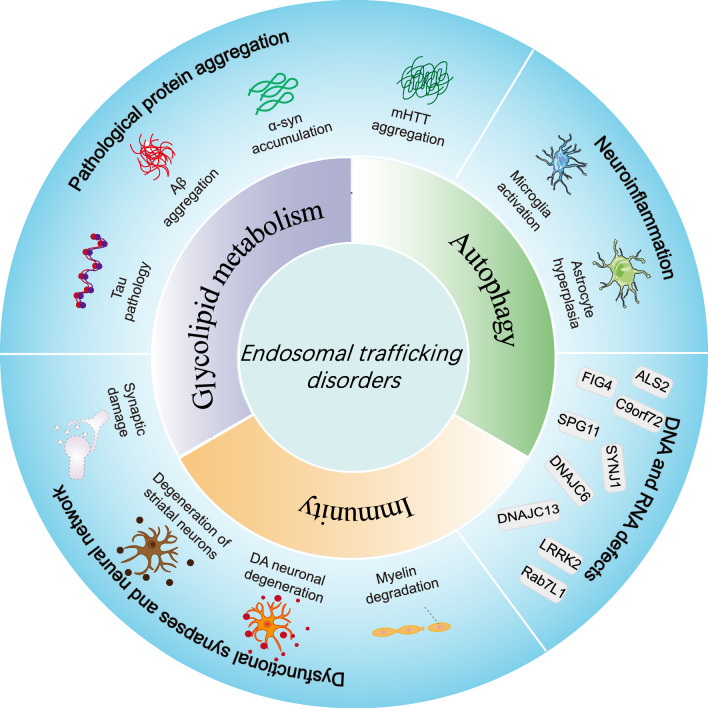

## Introduction

The intracellular membrane system constitutes a set of membranes and organelles that are structurally and functionally interrelated, encompassing the plasma membrane, the endoplasmic reticulum (ER), the Golgi apparatus, lysosomes, and endosomes. The transfer of materials between compartments is typically accomplished through vesicular transport. Vesicle transport starts from formation of a vesicle by budding from the membrane, and completes through fusion of vesicle and target membranes. Endosomes are membrane-wrapped vesicle structures implicated in intracellular sorting, mainly transporting receptors, lipid membranes, extracellular fluids, and other substances taken up through endocytosis. Endosomes serve as the initial destination of substances internalized by the plasma membrane and the principal sorting station in the endocytosis pathway [1–3]. Specifically, freshly synthesized proteins can reach endosomes via the Golgi apparatus or be transported to the endosomes by plasma membrane. Endocytosed molecules can be transported through endosomes to lysosomes for degradation or transported to the trans-Golgi network (TGN) or the plasma membrane for recycling [4]. Additionally, endosomes can participate in secretion by forming exosomes [5, 6]. Therefore, the equilibrium between lysosomal degradation and the efflux pathway mediated by endosomal transport is important for the homeostatic regulation of proteins and metabolites within cells.

Dysregulation of substance transport and metabolite clearance caused by aberrant endosomal transport is associated with the initiation and progression of neurodegenerative diseases (NDDs), including Alzheimer's disease (AD) and Parkinson's disease (PD) [7, 8]. In this review, we aim to overview the endosomal transport pathways and their regulators, as well as their involvement in physiological processes. We also explore the roles and potential mechanisms of endosomal transport disorders in the common pathologies of NDDs. Finally, we summarize potential drugs that can impact NDD pathology by regulating endosomal transport pathways, in order to provide new strategies for prevention and treatment of NDDs.

## Classification of endosomal trafficking routes and key regulators

Endosomes are acidic, lysosomal-free vesicles that can be classified into early endosomes, late endosomes, and recycling endosomes. Early endosomes are membrane-bound organelles containing endocytosed substances, having an acidic microenvironment that dissociates the internalized receptor-ligand complexes and cargoes for classifying them to the correct destinations. Early endosomes serve as the main sorting station and crossroad for internalized receptors and membranes [2, 9]. Markers for early endosomes include Rab5, Rab4, and EEA1 (early endosome antigen 1). As early endosomes move along the microtubule to the perinucleosome, they mature into late endosomes. Most of them fuse with lysosomes to form the endosomal-lysosomal compartment that offers an acidic environment for degradation of cargoes in the intraluminal vesicles (ILVs), often situated in the medial part of the cytoplasm, near the nucleus. The markers for late endosomes include Rab7, Rab9, and mannose 6-phosphate receptors. The recycling endosomes, a form derived from the tubular structure protruding from the surface of early endosomes, is involved in the cycling of cargo proteins from early endosomes to plasma membrane. The marker for recycling endosomes is Rab11 [10].

Serving as the core transport routes, endosomes can transfer diverse molecular cargoes between cell membrane and organelles as well as among organelles [11]. For newly synthesized proteins and lipids, a majority of them are transported out of the Golgi apparatus from the TGN, which functions as the initial site for cellular sorting. Subsequently, they are either transported to the endosomes or reach the plasma membrane, where they are captured by sorting receptors and internalized through the endocytotic pathway. Proteins and lipids located outside cells and on the cell membrane, can also be internalized through the plasma membrane into endosomes, and sorting begins after they enter the early endosomes. Proteins requiring recycling can be directly transported to the cell surface via the tubular vesicle transport route, a process also known as "rapid recovery". Receptors for recycling exit early endosomes and are directly transported to the plasma membrane [12], or enter the slow recycling pathway through the recycling endosomes, typically confined to the perinuclear recycling compartment. In addition, the TGN can also facilitate retrograde transport to the cell surface [13]. Other cargoes are sorted into late endosomes. During maturation of early endosomes into late endosomes, the membrane invaginates to form multivesicular bodies (MVBs) containing ILVs [14, 15]. The majority of MVBs fuse with lysosomes to form endolysosomes, where the ILVs and their associated cargoes are degraded [14].

**Fig. 1:**
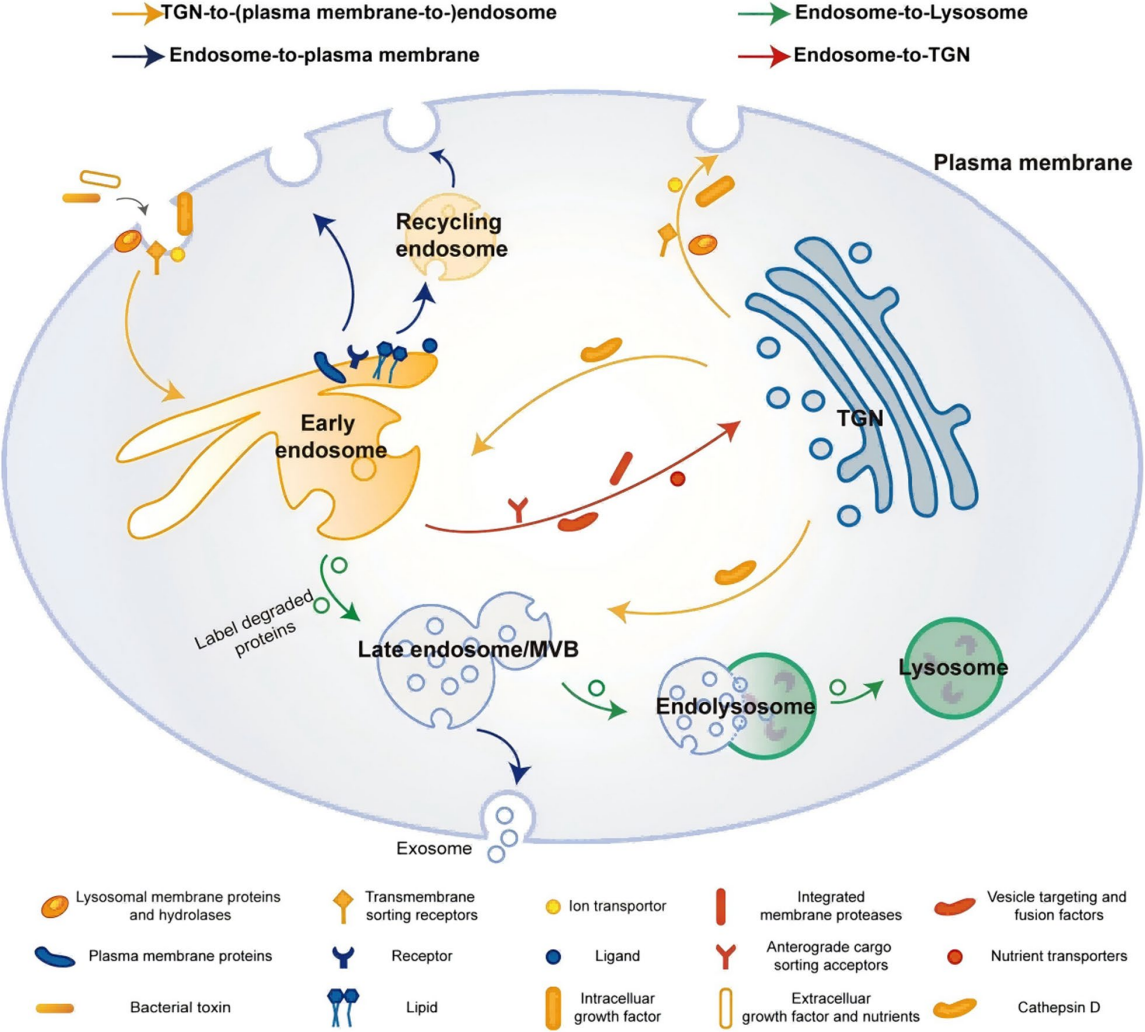
Schematic of endosome-related trafficking pathways and their transported cargoes

Endosomes also play a role in secretion by generating small vesicles known as exosomes. Apart from being delivered to lysosomes for processing and recycling, MVBs can also fuse with the plasma membrane to release ILVs into the extracellular environment to facilitate intercellular communication [5, 6, 15]. Markworth et al. have revealed that late endosomes can retrieve tropomyosin receptor kinase TrkA through tubular microdomains. The neurotrophic signaling from Trks are essential for synaptic formation, axon growth, and neuronal survival. Specifically, endophilinAs and WASH1 are involved in the formation of tubular structures in early endosomes and interact with TrkA to facilitate TrkA retrieval by late endosomes and MVBs. This process enables the transportation of neurotrophic receptors from synapses to cell bodies [16]. In conclusion, the endosome-centered transport pathways encompass the TGN-(to-plasma membrane-)to-endosome route, the endosome-to-lysosome route, the endosome-to-plasma membrane cycling route, and the endosome-to-TGN retrograde transport route (Fig. 1). These molecular processes play a crucial role in determining the destiny of protein and lipid degradation or recycling, and are essential for maintaining intracellular homeostasis of proteins and lipids.

Numerous regulators participate in endosome pathways, including the Rab proteins, ADP ribosylation factors (ARFs), the adaptor protein (AP) complex, and ADP-ribosylation factor-binding proteins (GGAs) (Table 1). Rabs are markers that define membrane identity and major regulators for vesicle formation, transportation, docking, and fusion with target membranes [17]. The ARF family consists of six ARF proteins, Sar proteins, and over 20 ARF-like proteins. ARFs play a key role in recruiting coat proteins and complexes during formation of transport vesicles, particularly at the Golgi apparatus [18, 19]. APs and GGAs serve as the primary effectors of ARFs. APs localize to the membranes of TGNs and/or endosomes in an ARF-dependent manner, and recruit clathrin and other accessory proteins by binding to sorting signals on their domains, concentrating cargo proteins into vesicles for intracellular transport. GGAs are ARF-dependent monomeric clathrin adaptor proteins, which are involved in the formation of clathrin-coated vesicles. During endosomal trafficking, numerous proteins individually or synergistically regulate the transport of cargo proteins in one or more routes.

**Table 1:** Endosome transport proteins and functions

Protein	Function	Subtype	Trafficking pathway	Animals/cells	References
Rab family	Intracellular transport of vesicles and membranes	Rab4	Endosome-to-plasma membrane	MDCK cells	[66]
		Rab5	Plasma membrane-to-endosome	HeLa cells	[263]
		Ypt6 (yeast homolog of Rab6)	Endosome-to-TGN	Saccharomyces cerevisiae	[264]
		Rab7	Endosome-to-lysosome	HeLa cells/ Rat primary hippocampal neurons	[50, 265]
		Rab8	Endosome-to-plasma membrane	Polarized MDCK cell/Rab8-deficient mice	[27, 68]
		Rab9	Endosome-to-TGN	CHO1021 cells	[266]
		Rab11	Endosome-to-plasma membrane	MDCK cells	[67]
		Rab14	TGN-to-plasma membrane-to-endosome-to-plasma membrane	NRK cells	[69, 267]
		Rab25	Plasma membrane-to-endosome-to-plasma membrane	MDCK cells	[67]
		Rab30	Endosome-to-TGN	HeLa cells	[268]
		Rab31	Plasma membrane-to-endosome	HeLa cells	[269]
ARF family	Recruit coat proteins and complexes during membrane transport vesicle formation	ARF1	TGN-to-plasma membrane	BHK-21 cells	[22]
		ARF4	TGN-to-plasma membrane-to-endosome	PNS enriched in photoreceptor biosynthetic membranes	[26]
		ARF5	Plasma membrane-to-endosome	HeLa cells	[31]
		ARF6	Endosome-to-plasma membrane	HeLa cells	[70]
		Arl1	Endosome-to-TGN	HeLa cells/BY4741 Yeast strain	[270, 271]
		Arl3p	TGN-to-plasma membrane	HeLa cells	[272]
		Arl5	Endosome-to-TGN	HeLa cells/COS-7 cells	[273, 274]
		Arl8	Endosome-to-lysosome	U937 cells	[275]
AP family	Concentrate the cargo protein sorting signal into vesicles for transportation by binding to it	AP-1	TGN-to-plasma membrane-to-endosom/Endosome-to-TGN/Endosome-to-plasma membrane	HeLa cell/Polarized MDCK cells	[23, 27]
		AP-2	Plasma membrane-to-endosome	HeLa cells	[276]
		AP-3	Endosome-to-lysosome	fibroblasts from embryonic	[277]
		AP-4	TGN-to-endosome	MDCK cell/MelJuSo cell lines	[25]
		AP-5	Endosome-to-lysosome	Human-derived fibroblasts	[278]
ESCRT	Promotes degradation of membrane proteins tagged by ubiquitin	ESCRTs-0	Endosome-to-lysosome	Rosetta pLys cells	[279]
		ESCRTs-I	Endosome-to-lysosome	Yeast cells	[280]
		ESCRTs-II	Endosome-to-lysosome	Yeast cells	[281]
		ESCRTs-III	Endosome-to-lysosome	Rosetta pLys cells	[282]
		CHMP2B	Endosome-to-lysosome	SK-N-SH cell lines	[58]
		CHMP3	Endosome-to-lysosome	HeLa cells	[59]
		CHMP5	Endosome-to-lysosome	Primary embryonic cells derived from E8.5 Chmp5^−/−^ embryos	[60]
Bin1	Dynamic remodeling of cell membranes		Endosome-to-plasma membrane	HEK293 cells	[164]
			Endosome-to-lysosome	N2a and HeLa S3 cells	[159]
CD2AP	Dynamic actin remodeling and membrane exchange		Endosome-to-lysosome	Primary cortical neurons	[160]
GGA family	Participate in the formation of CCV	GGA1	TGN-to-plasma membrane-to-endosome	HeLa cells	[28]
		GGA2	TGN-to-plasma membrane-to-endosome	HeLa cells	[23]
		GGA3	TGN-to-plasma membrane-to-endosome	A7 cell/HeLa cells	[92]
Retromer complex	Recycles proteins for retrograde transport	VPS26, VPS29, VPS35	Endosome-to-TGN	HeLa cells	[84]
		SNX 1/2/5/6	Endosome-to-TGN	HeLa cells	[85, 86]
		SNX8	Endosome-to-TGN	HeLa cells	[89]
		SNX17	Endosome-to-plasma membrane	HeLa cells	[73]
		SNX27	Endosome-to-plasma membrane	HEK 293 cells/A10 cells	[74]
SORL1	An adaptor molecule of retromer complex		Endosome-to-TGN	HEK293 cells	[90]
PACS1	TGN membrane protein localization		TGN-to-plasma membrane-to-endosome	A7 cell/HeLa cells	[92]
SYNJ1	Phosphatase acting on various phosphoinositides		Plasma membrane-to-endosome/ Endosome-to-lysosome	*Synj1*^+/−^ mice/Transgenic zebrafish	[53, 283]
LRRK2 complex			Endosome-to-plasma membrane	Mouse embryonic fibroblasts	[75]
DNAJC protein family	Plays a critical role in protein folding	DNAJC13	Endosome-to-plasma membrane	COS7 cells	[76]
		DNAJC5	Endosome-to-plasma membrane	SH-SY5Y cells	[80]
		DNAJC6	Plasma membrane-to-endosome	DNAJC6 knockout mouse	[33]

## TGN-(to-plasma membrane-)to-endosome trafficking

A main function of the Golgi apparatus is to process, sort, and transport proteins and lipids synthesized in the ER. Cargoes such as transmembrane sorting receptors, ion transporters, lysosomal membrane proteins and hydrolases are transferred from TGN as a starting point for sorting and transport, to endosomes. This process is crucial for the delivery of lysosomal hydrolases to the degradation pathway, as well as for biosynthesis and transport. TGN-derived cargoes can be transported to the plasma membrane and subsequently internalized into early endosomes. Molecules such as cathepsin D can also be directly transported to the endosomes [20]. Clathrin-mediated endocytosis is the primary pathway for internalizing the plasma membrane components. During this process, specific motifs in the cytoplasmic domain of transmembrane proteins are recognized by a protein-coated pit, leading to rapid internalization into early endosomes [21]. This mechanism is essential for physiological processes such as nutrient uptake, immune response, and cell surface receptor signaling transduction [11]. Following internalization, the endocytic substances are targeted to a series of intracellular compartments via endosomal sorting, thereby entering the degradation or circulation pathway.

ARF1 is a primary ARF protein involved in the Golgi apparatus sorting of cargoes. ARF1 is predominantly localized in the Golgi apparatus and regulates vesicle budding and uncoating within the Golgi complex [22]. Activated ARF1 can recruit AP-1, AP-3, and AP-4 to TGN, facilitating their association with the Golgi apparatus for cargo collection and regulating the TGN-to-plasma membrane-to-endosomal transport route [23–25]. ARF4 controls vesicle budding from the TGN [26]. In addition, by synergizing with Rab8, AP-1B can control the transport of newly produced membrane proteins to the basolateral surface of epithelial cells [27]. Nonetheless, GGAs can participate in the formation of clathrin-coated vesicles through interactions with AP-1, thereby contributing to the TGN-to-plasma membrane-to-endosomal trafficking pathway. There are two modes of GGA participation: first, GGA2 relies on AP-1 for clathrin-coated vesicle formation; second, GGA1/3 collects the cargo on the membrane and transfers it to AP-1 for final packaging into clathrin-coated buds [23, 28].

ARF6 and AP-2 play key roles in internalization from the plasma membrane to endosomes. ARF6 is the sole ARF protein exclusively localized to the plasma membrane, and its active form facilitates AP-2 recruitment to the plasma membrane, thereby initiating endocytosis [29, 30]. A previous study showed that another member of the ARF family, ARF5, mediates integrin internalization, suggesting a role in clathrin-mediated endocytosis of specific cargoes [31]. Furthermore, Rab5, a key marker of early endosomes, regulates the internalization and trafficking of membrane receptors by controlling vesicle fusion and receptor sorting within early endosomes, thereby playing a crucial role in the endocytic trafficking of neurotrophic factors [32]. Auxilin from the DNAJC protein family and synaptojanin 1 are involved in the endocytosis from the plasma membrane to endosomes by facilitating the shedding of the clathrin coat from clathrin-coated vesicles [33–35].

## Endosome-to-lysosomal trafficking pathway

Proteins that are internalized into early endosomes are often transported by late endosomes into lysosomes for degradation, a process that requires specific targeting information, i.e., ubiquitination modifications. Lysosome-targeted cargoes are first modified by monoubiquitination or polyubiquitination at lysine residues in the cytoplasmic domain. Then the endosomal sorting complex required for transport (ESCRT) machinery sorts the ubiquitinated cargoes, initiating multiple rounds of cargo sorting, enrichment, and ILV biogenesis to allow efficient delivery to lysosomes [36–38]. Late endosomes then fuse with lysosomes to form endolysosomes, in which ILVs and the cargoes they carry are degraded. At this stage, the lysosomes may undergo fusion with other organelles, such as autophagosomes, for the degradation and clearance of organelles or proteins [39]. In addition, non-ubiquitin-dependent pathways for targeting cargoes to ILVs have also been identified, and ESCRT-independent biogenesis of ILVs in endosomes has also been reported [40–42]. In conclusion, the endosome-to-lysosomal transport route is important for biological processes such as nutrient uptake, immunity, and signal transduction, and its perturbation may lead to abnormal retention and accumulation of cargoes or pathogenic proteins in mature endosomes, leading to lysosomal dysfunction and cell death [43, 44].

The ESCRT machinery is essential for MVB biogenesis [45]. ESCRT comprises distinct polymeric protein complexes (ESCRTs-0, -I, -II, and -III), which are successively recruited from the cytoplasm to the endosomal membrane to facilitate the sorting of ubiquitinated proteins and ILVs, thereby generating MVBs [46]. Subsequently, MVBs undergo fusion with lysosomes, while ILVs are degraded within the lysosomal lumen. Furthermore, Rab7, a marker of late endosomes, replaces Rab5 during endosome maturation [47]. Rab7 is essential for endosomal maturation, transport of proteins from endosomes to lysosomes, transport within endosomal and lysosomal compartments, fusion between late endosomes and lysosomes, lysosomal biogenesis, and intracellular transport of autophagosomes, thereby regulating protein turnover and maintaining protein homeostasis [48–50]. In *Drosophila*, protein ema, the human C-type lectin protein 16A (CLEC16A) ortholog, is directly involved in endosomal maturation. In ema mutant *Drosophila*, both early and late endosomes fail to develop into mature MVBs and lysosomes [51]. In addition, Synaptojanin 1 also plays an important role in the endosome-lysosomal transport pathway, and its defects lead to late endosomal abnormalities and autophagic destruction [52, 53].

The HOPS (homotypic fusion and vacuole protein sorting) complex plays a crucial role in the endosome-lysosomal docking process (also known as the tethered process). Overexpression of its components, vacuolar protein sorting 18 (VPS18) and VPS39, results in abundance of late endosomes and lysosomes [54, 55]. As a component of the trans-SNARE (soluble NSF (N-ethylmaleimide-sensitive factor) attachment protein receptor) complex, vesicle-associated membrane protein 7 (VAMP7) facilitates endosomal-lysosomal membrane fusion. The chaperone AP-3, which binds to VAMP7, is also essential for proper delivery along the endosome-lysosomal transport route [56]. Deficits in AP-3 in both humans and mice lead to impairments in the endosome-lysosomal sorting pathway [57]. Additionally, Bin1, CD2AP, as well as the ESCRT proteins charged multivesicular body protein (CHMP)2B, CHMP3, and CHMP5, contribute to the process of endosome-lysosomal fusion [58–60].

Apart from the aforementioned essential regulators, ARFs and GGAs are also implicated in the control of endosome-lysosomal trafficking pathways. Arl8 facilitates the transfer of endocytic macromolecules to lysosomes and regulates lysosome motility [61]. GGAs can bind ubiquitin and participate in the trafficking of endosomal ubiquitinated cargoes to lysosomes [62].

## Endosome-to-plasma membrane trafficking cycle

Before early endosomes translocate to the perinuclear region, membrane proteins and lipids need to be efficiently cycled back to the plasma membrane in a precise manner to ensure sustained endocytic uptake. Thus, during initial stages of endosomal circulation, recyclable cargoes should avoid entering ILVs for degradation. Through the "rapid recovery" pathway, these recyclable molecules are sorted and transported back to the plasma membrane via tubular-vesicular carriers [12, 63]. The primary sorting mechanism in early endosomes is "geometry-based sorting" rather than the identification of specific sorting motifs in cargo proteins [12]. In this process, due to the larger surface area-to-volume ratio of the tubular membrane compared to that of the vesicular portion of early endosomes, most membrane proteins and lipids are sorted from the cargoes by pinching off of the tubules [64, 65].

In mammalian cells, Rab4, Rab8, Rab11, and Rab14 are involved in the endosome-plasma membrane cycle pathway. Various factors may govern the trafficking of membrane proteins and lipids to distinct areas of the cell surface. For example, mutations in Rab4 result in the relocalization of transferrin receptors from basolateral to apical plasma membrane of epithelial cells. Recycling to the basolateral surface is mediated by Rab8 and Rab11, whereas Rab8 and Rab14 may be involved in the apical targeting pathway [27, 66–69]. Rab25 also regulates the transcytosis of immunoglobulin G [67].

ARF6 targets recycling endosomal vesicles to the plasma membrane by localizing on endosomes and the plasma membrane in a GTP-dependent manner [70]. Additionally, retromer complexes mediate the endosome-to-plasma membrane cycle path to retrieve and deliver membrane proteins, such as glucose transporter (GLUT)1, Sortilin, and cation-independent mannose-6-phosphate receptor (CI-MPR), directly to the cell surface [71]. Specifically, SNX3 facilitates the recycling of transferrin receptors; SNX17 regulates integrin recovery that shifts integrins away from degradation pathways, supporting cell migration; and SNX27 acts as an adapter that binds to cargoes containing PDZ ligands to regulate β−2 adrenergic receptor transport from endosomes to the plasma membrane [72–74].

The leucine-rich repeat kinase 2 (LRRK2) complex and members of the DNAJC protein family (DNAJC13 and DNAJC5) are also involved in regulating the endosome-to-plasma membrane transport route. The LRRK2 complex negatively regulates Notch signaling by modulating the endosome-to-plasma membrane trafficking of the Notch ligand Delta-like 1 (Dll1)/Delta (Dl), thereby accelerating neural stem cell differentiation and regulating the function and survival of differentiated dopaminergic neurons [75]. DNAJC13 (also known as receptor-mediated endocytosis-8, RME-8), localized in early endosomes and recycling endosomes, regulates the endosome-to-plasma membrane transport of transferrin [76]. DNAJC13 also plays an important role in endosomal trafficking. It is recruited to early endosomes through interaction with phosphatidylinositol 3-phosphate (PtdIns3P) [77]. Deletion of DNAJC13 leads to the accumulation of clathrin in early endosomes and the misclassification of retromer-dependent cargoes [78, 79]. In contrast, DNAJC5 can control the transfer of NDD-associated pathological proteins to endosomes and their subsequent release into the plasma membrane [80].

## Endosome-to-TGN retrograde trafficking

In addition to the rapid endosome-to-plasma membrane transport pathway, cargoes can also be recycled to the plasma membrane via the endosome-to-TGN retrograde pathway, which is one of the primary routes for transporting proteins and lipids away from lysosomal degradation. This process regulates various physiological and pathological processes such as the early development of metazoans, cell homeostasis, retrograde sorting in neurons, and toxin invasion. Protein cargoes received via retrograde transport include bacterial toxins (e.g., Shiga toxin) that enter cells through retrograde pathways and cause cytotoxicity, integrated membrane proteases, vesicle targeting and fusion factors (e.g., SNAREs), nutrient transporters, and anterograde cargo sorting receptors that are depleted from TGN during anterograde transport [81].

The retromer complex plays a pivotal role in the endosome-to-TGN retrograde trafficking. Its recruitment from retrosomes to the endosomes and its role in tubulation formation are essential for early retrograde transport of cargoes to TGN [82, 83]. The retromer complex consist of VPSs and sorting nexins (SNXs). VPS26, VPS29, and VPS35 form a trimeric subcomplex that mediates cargo recognition during cargo protein transport, while the SNX 1/2/5/6 components control tubule formation [84–86]. In the VPS26-VPS29-VPS35 trimeric subcomplex, VPS35 is the core molecular framework acting as a scaffold for the binding of all other retromer monomeric modules and retromer receptors [87]. The trimeric subcomplex collectively selects and binds to the retrograde transport cargoes, facilitating their packaging into tubular carriers via SNX 1/2/5/6 localized in the early endosomes, thereby mediating retrograde transport from early endosomes to TGN [88]. In addition, SNX8 is involved in the transport of Shiga toxins (classic bacterial toxins) from endosomes to TGN [89]. SORL1 (also known as SORLA) functions as an endosomal sorting protein and a connector for retrosomal transport, enabling transport from endosomes to the Golgi apparatus in a retromer-dependent manner [90].

The endosome-to-TGN retrograde trafficking is regulated by Rab, ARF, and AP protein family members (Table 1). CLEC16A is involved in the retrograde transport of compartments containing human leukocyte antigen II (HLA-II) in bone marrow cells [91]. In addition, phosphofurin acidic cluster sorting protein 1 (PACS1) and EHD1 (Eps15 homology domain-containing protein-1) also participate in the endosome-to-TGN retrograde transport of cargo proteins. The former eliminates GGA3 to direct endosome-to-TGN retrieval of CI-MPR, while the latter stabilizes SNX1-positive tubules to promote trafficking from endosomes to the Golgi apparatus [92, 93].

## Effects of endosomal trafficking disorders on physiological processes

Noxious stimuli, such as overactivation of inflammasomes or aggregation of disease-related toxic proteins, can lead to endosomal trafficking disorder, characterized by increased endosomal volume and quantity, as well as changes in the expression levels of endosomal transport regulators [94]. Consequently, the balance of cargo entry in and exit from endosomes is disrupted, leading to their retention within cellular compartments. This further results in disruptions of physiological processes such as glycolipid metabolism, autophagy, and immunity.

### Endosomal transport defects and glycolipid metabolism

**Fig. 2:**
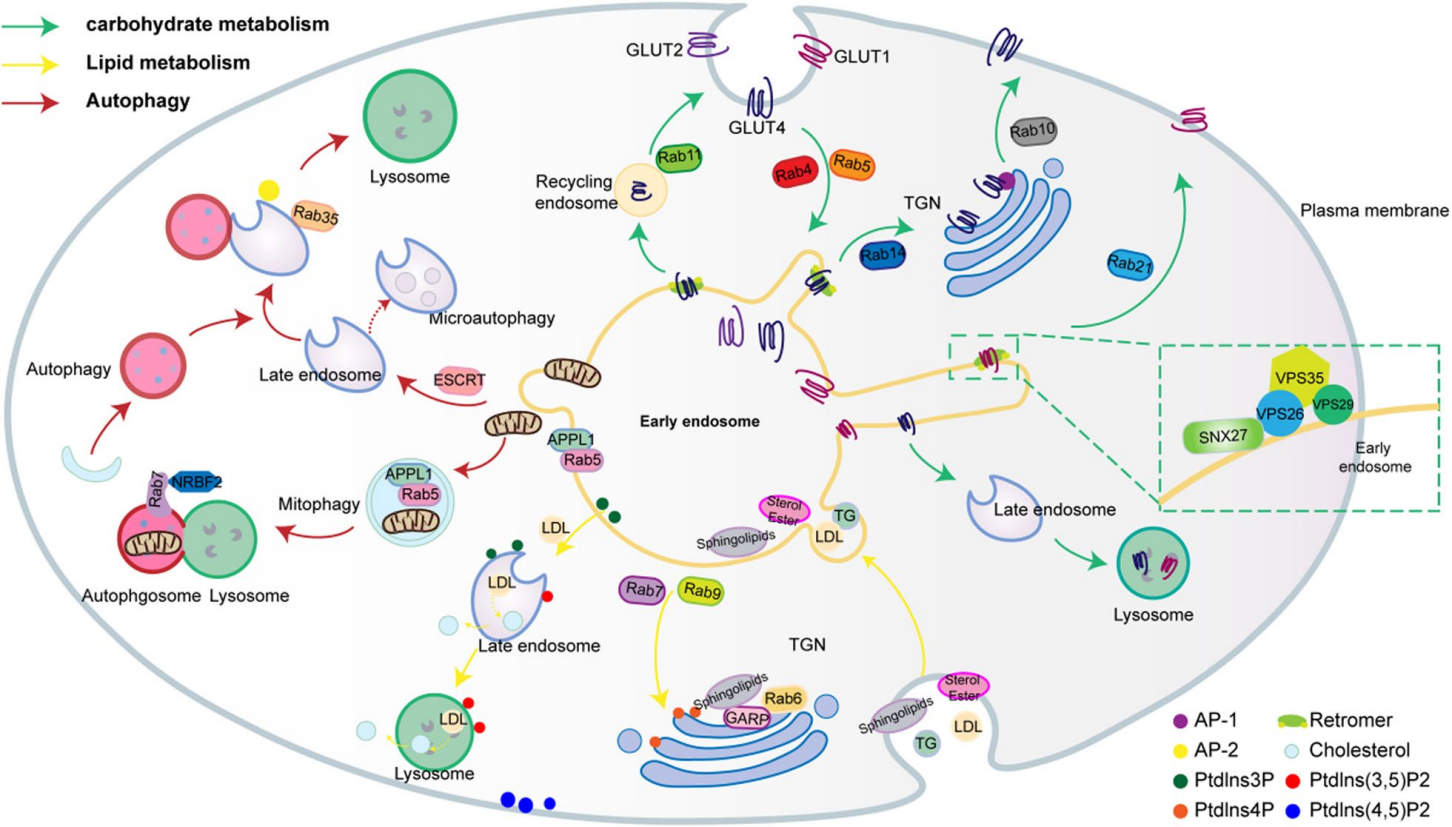
Endosomal transport pathways interact with glycolipid metabolism and autophagy. Endosomal trafficking pathways regulate carbohydrate metabolism by modulating the spatial distribution of GLUTs (green arrows); endosomal trafficking pathways sort and degradate internalized lipids, with phosphatidylinositol regulating these routes (yellow arrows); endosomal trafficking pathways are involved in autophagy or mitophagy through endosomal microautophagy or by controlling the fusion between autophagosomes and lysosomes (red arrows)

Glucose and lipids are vital suppliers of substrates and energy for organisms. The homeostatic balance of glycolipid metabolism is an important guarantee for organisms to cope with changes in the internal and external environments to maintain normal physiological function. The endosomal transport pathway plays a pivotal role in the uptake and decomposition of glucose and lipids (Fig. 2).

#### Carbohydrate metabolism and endosomal trafficking defects

The endosomal transport system contributes to cellular glucose uptake by adjusting the spatial distribution of GLUTs. The expression of GLUT1, GLUT2, and GLUT4 on the surface of the plasma membrane and their internalization rate determine the uptake efficiency of glucose. In mammals, the insulin-dependent translocation of GLUT4 tightly regulates glucose levels. GLUT4 resides in subcellular compartments such as endosomes and TGNs in basal adipocytes. Upon insulin stimulation, GLUT4 translocates to the plasma membrane through Rab11-mediated endosomal-to-plasma membrane circulation and TGN-to-plasma membrane trafficking mediated by Rab10 and AP-1 [95]. Rab4 and Rab5 mediate the internalization of GLUT4. Following internalization, in addition to recycling back to the plasma membrane, GLUT4 can also be recycled to the TGN in response to Rab14 and retromer complexes [96–98]. If the process of retrieving GLUT4 from endosomes is compromised, GLUT4 will be assigned to the endosome-lysosomal degradation pathway. Furthermore, SNX27 as a component of the retromer complex, promotes the transport of transmembrane receptors such as GLUT1 from endosomes to the plasma membrane. PTEN, a tumor suppressor, blocks the association of SNX27 with VPS26, thereby inhibiting assembly of the retromer complex, resulting in reduced cellular glucose uptake due to impaired recycling of GLUT1 from endosomes to the plasma membrane [99]. Depletion of Rab21, a member of the Rab5 subfamily, inhibits the endosomal-to-plasma membrane transport mediated by the SNX27-retromer complex, leading to impaired glucose uptake [100].

An essential function of the endosome-to-lysosomal transport route is to control the metabolic signaling of the glucagon receptor (Gcgr), which is involved in the regulation of glucose and lipid metabolism. VPS37a, a member of the ESCRT-I complex, controls the intracellular transport pathway of Gcgr within endosomes and lysosomes. Reduction in VPS37a level leads to Gcgr accumulation in early endosomes and decreased localization within lysosomes, leading to activation of hepatic glucose production without affecting fatty acid β oxidation [101]. Additionally, ESCRT negatively regulates Erg6 turnover by monitoring its localization and deterioration as a marker for lipid droplets [102]. Ding et al. observed that the myeloid-derived suppressor cells (MDSCs) from lysosomal acid lipase-deficient (lal-/-) mice exhibited increased expression of Rab7, a crucial enzyme that controls the endosome-to-lysosomal transport system. Through direct interaction with mTOR, Rab7 regulates its activity, thereby influencing metabolic reprogramming in MDSCs. This leads to reduced glucose consumption and increased healthy mitochondria [103]. Furthermore, metabolic disturbances can also interfere with endosomal trafficking; for instance, glucose starvation triggers a transcriptional response that increases internalization from the plasma membrane to endosomes in yeast [104].

#### Lipid metabolism and endosomal trafficking defects

The plasma membrane-to-endosome transport route is also responsible for cellular lipid uptake. This pathway allows the entry of several lipids into cells, including sterol esters, sphingolipids, triglycerides, and low-density lipoprotein (LDL) cholesterol. Endosomes play a role in lipid sorting as well. For example, sphingolipids are targeted to the Golgi apparatus through the retrograde endosome-to-TGN route via Rab7 and Rab9 [105], while Rab6 is necessary for limiting the GA-related retrograde protein transport (GARP) complex to dock with the Golgi apparatus [106]. Moreover, the endosome-to-lysosomal transport system plays a crucial role in lipid catabolism. Hepatocytes aggressively remove LDL through endocytosis, and the cholesteryl esters from the internalized LDL are hydrolyzed into free cholesterol within late endosomes or lysosomes. Subsequently, cholesterol is further transferred to other cellular compartments for incorporation into intracellular membranes or metabolized into bile acids and released into the biliary duct [107].

Lipids, as the primary constituents of endocytic vesicles, also participate in the regulation of endosomal trafficking routes. Phosphoinositide acts as a membrane signaling platform and plays a pivotal role in coordinating lipid metabolism. In the TGN-to-plasma membrane-to-endosome transport pathway, phosphatidylinositol 4-phosphate (PtdIns4P) is essential for the formation of plasma membrane-targeting vesicles in TGN [108]. PtdIns4P participates in the membrane sensing process by promoting recruitment of four-phosphate adaptor protein (FAPP) 1, FAPP2 and ARF1, which are essential for glycosphingolipid metabolism, to TGN [109]. PtdIns4P is also necessary for receptor sorting at early endosomes [110]. Conversely, PtdIns3P preserves ubiquitinated receptors on early endosome membranes and subsequently transfers them to ESCRT to facilitate receptor incorporation into MVBs [111]. Furthermore, PtdIns(4,5)P2 (phosphatidylinositol-4,5-bisphosphate), located on the plasma membrane, recruits multiple effector proteins required for endocytosis by interacting with ENTH (Epsin N-terminal homology), ANTH (AP180 N-terminal homology), or PH domain to participate in the plasma membrane-to-endosome transport [112]. PtdIns(3,5)P2 (Phosphatidylinositol-3,5-bisphosphate), enriched in late endosomes/MVBs, acts as a regulator of endosome-to-lysosomal transport [113].

### Endosomal transport defects and autophagy

Autophagy is a lysosome-mediated biological process that facilitates the degradation of damaged cytoplasmic proteins, organelles, and invading microorganisms to fulfill the metabolic requirements of cells, the renewal of certain organelles, and the response to extracellular stimuli. Autophagy is indispensable for maintaining cellular homeostasis (Fig. 2).

Endosomes can directly participate in autophagy through endosomal microautophagy, which refers to the invagination of late endosomal membranes that generates MVBs for direct phagocytosis and degradation of inclusions. This process is an essential mechanism for sorting and turnover of membrane-related presynaptic proteins in mature synapses [114]. The endosome-to-lysosomal transport pathway is involved in the physiological process of autophagy by participating in the transport of autophagosomes. Autophagosomes are generated at synaptic terminals in neuronal cells, while lysosomes are enriched in cell bodies. The autophagosomes transport retrogradely from synaptic terminals along microtubules to the soma by fusing with late endosomes, and during retrograde movement, autophagosomes fuse with lysosomes to acidify and degrade their cargoes [39]. This process is crucial for the continuous renewal and functional maintenance of synaptic terminal membrane proteins, particularly synaptic vesicle proteins [115, 116]. Proteins involved in endosomal transport, such as AP-2, Rab35, and ESCRT complexes, are involved in the retrograde transport of autophagosomes [116, 117]. In addition, early endosomes regulate autophagy and mitophagy under stress, while APPL1 and its interacting partner Rab5 promote endosome-mediated mitophagy [118, 119]. The autophagic protein NRBF2 interacts with Rab7 to promote autophagosome maturation, thereby attenuating neuroinflammation and oxidative stress [120].

Autophagy is also involved in endosomal transport disorders. Impairments in phagocytosis mediated by the microglial autophagy protein Beclin 1 are associated with impaired recruitment of retromers to the phagocytic membrane and decreased levels of retromer monomers [121]. CLEC16A, which regulates autophagy and mitophagy, closely interacts with retromer components to control endosomal fate [122].

### Endosomal transport defects and immunity

**Fig. 3:**
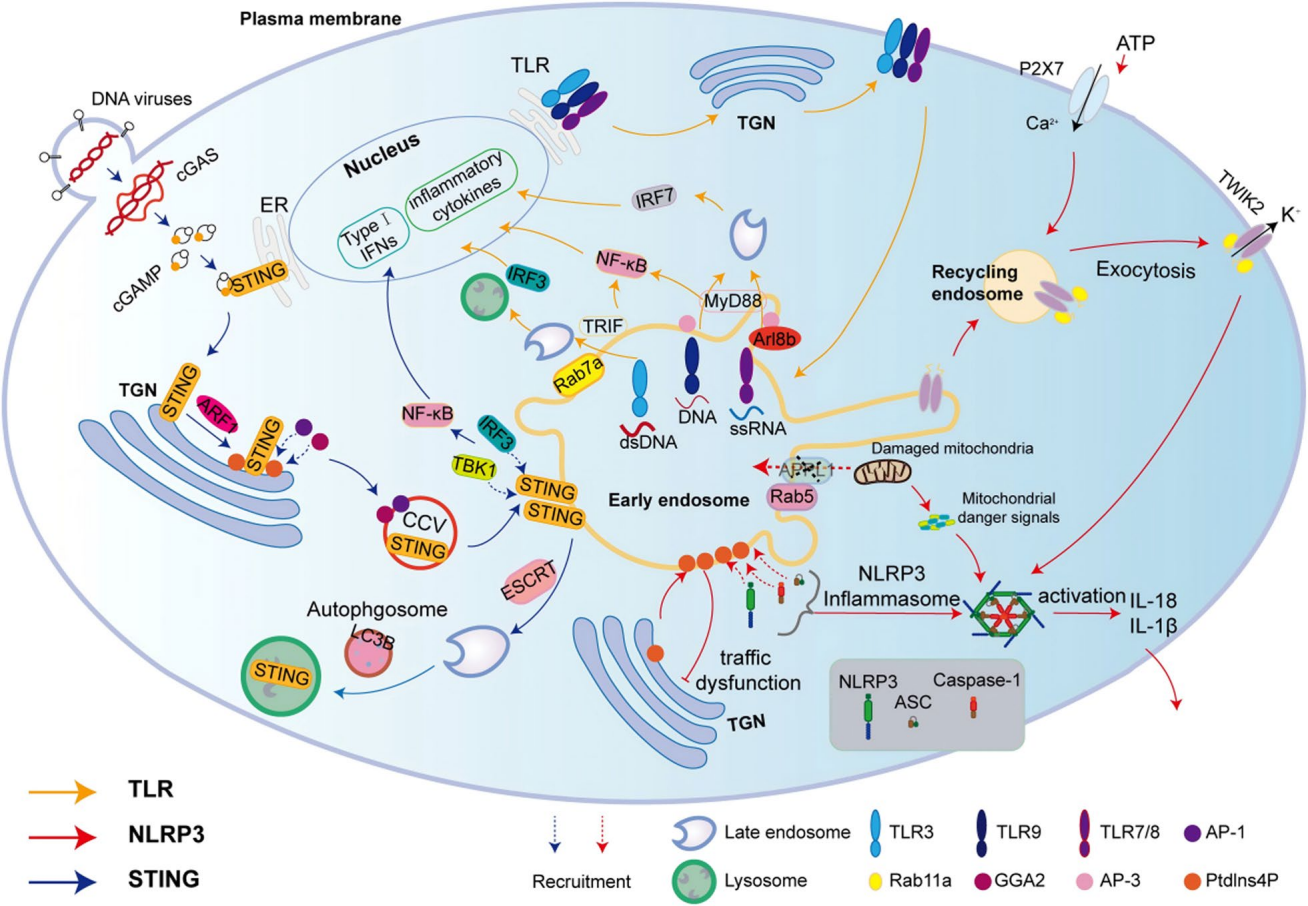
Endosomal transport pathways modulate immunity by regulating activation of TLRs, NLRP3 inflammasomes, and STING signaling. Endosomal transport lines regulate the activation of TLR3, TLR7/TLR8, and TLR9 (yellow arrows); PtdIns4P clustering within endosomes, the plasma membrane translocates of TWIK2, and impaired mitochondrial autophagy activate NLRP3 inflammasomes (red arrows); endosomal trafficking pathways control the activation and termination of STING signaling (blue arrows)

Multiple pathogens invade and control endosomal compartments as a survival strategy within host cells. Endosomes serve as the site for pathogen sensing and for processing and loading of antigens to major histocompatibility complex molecules [123]. Endosomal trafficking pathways play a crucial role in controlling the body's response to bacterial and viral infections, specifically involving the activation of Toll-like receptors (TLRs), the NOD-like receptor thermal protein domain associated protein 3 (NLRP3) inflammasome, and stimulator of interferon gene (STING) signaling (Fig. 3).

#### TLRs and endosomal trafficking

TLRs are a class of pattern recognition receptors present on macrophages and dendritic cells that mediate recognition and response to distinct pathogen-associated molecular patterns (specific substances released by invading bacteria and viruses), thereby playing a pivotal role in immune cell survival, proliferation, and inflammation.

After synthesis and maturation in the ER, TLRs are transported out of the ER and into endosomes via the TGN-plasma membrane-endosome pathway. TLR members such as TLR3, TLR7, TLR8, and TLR9 are located within endosomes, where a lower pH level is required for their ligand recognition and signal transduction. These receptors can sense viral, bacterial, and self-derived nucleic acids. The compartmentalization of TLRs in endosomes restricts their activation by self-derived nucleic acids and diminishes the likelihood of an autoimmune response. The responsiveness of these TLRs is controlled by their expression, distribution, and proteolysis in the endosomal compartments. Extracellular and intracellular DNA and RNA are transported to endosomal compartments, where DNase and RNase degrade them into nucleosides. TLR3, TLR7/8, and TLR9 are able to recognize degradation products. The ligand-activated TLRs subsequently initiate endosome-lysosomal trafficking, leading to the alteration of downstream signaling pathways from the NF-κB signaling pathway to the interferon regulatory factor (IRF) signaling pathway [124]. In this transport route, TLR3 activation initiates Rab7-dependent transport, TLR7/8 activation promotes Arl8b- and AP-3-dependent transport, and downstream signal transport after TLR9 activation requires the participation of AP-3 [125–127].

#### NLRP3 inflammasome activation and endosomal trafficking disorders

The NLRP3 inflammasome is a cellular multiprotein complex composed of NLRP3 protein, procaspase-1, and ASC. The NLRP3 inflammasome can recognize pathogen-associated molecular patterns and damage-associated molecular patterns, resulting in activation of caspase-1. Activated caspase-1 further facilitates the maturation and secretion of inflammatory mediators, such as IL-1β and IL-18, which can block phagocytosis, trigger innate immune responses, and play a pivotal role in host defense against infectious agents. NLRP3 activation has been shown to lead to disruptions in endosomal trafficking [128].

Disorders of endosomal trafficking, involving plasma membrane-to-endososome, endosome-to-TGN, and endosome-to-plasma membrane transport routes, also affect NLRP3 inflammasome activation. First, the neuropeptide calcitonin gene-related peptide (CGRP) can be internalized into early endosomes after secretion. Once NLRP3 inflammasome stimulation triggers endosome leakage, CGRP is released into the cytosol and directly binds to NLRP3, destabilizing the NLRP3-NEK7 complex, thereby inhibiting the NLRP3 inflammasome activation and exacerbating bacterial infection [129]. Second, under normal conditions, the abundance of PtdIns4P on endosomes is minimal; however, disruption of endosome-to-TGN transport leads to excessive PtdIns4P accumulation on endosomes. As phosphoinositides function to recruit NLRP3 to endosomes to initiate the inflammasome activation process [130], the PtdIns4P accumulation results in NLRP3 recruitment and enhanced NLRP3 inflammasome activation [131]. Finally, proper functioning of the endosome-to-plasma membrane cycling pathway is also of great significance for the NLRP3 inflammasome activation. The NLRP3 inflammasome cannot be activated without potassium efflux through the potassium channel, the two-pore domain weak inwardly rectifying K^+^ channel 2 (TWIK2). The endosome-to-plasma membrane transport carries endosomal TWIK2 to the plasma membrane in response to an increase in extracellular ATP to facilitate potassium excretion. Defects in Rab11a, which regulates the endosome-to-plasma membrane transport route, reduce TWIK2 translocation to the plasma membrane in macrophages, thus inhibiting NLRP3 inflammasome activation [132]. In addition to the aforementioned endosomal transport barriers that directly affect NLRP3 inflammasome assembly and activation, APPL1 and Rab5 endosomes support the delivery of damaged mitochondria for mitophagy, preventing NLRP3 inflammasome activation by mitochondria-derived danger signals [119, 133].

#### STING signals and endosomal trafficking

The STING protein functions as an in vivo pattern recognition receptor for innate immune responses triggered by cyclic dinucleotides, typically inducing the expression of type I interferon by dendritic cells. Activation of STING requires the involvement of cGAS (cyclic GMP-AMP synthase), which recognizes exogenous DNA and produces cyclic GMP-AMP (cGAMP), a ligand for STING. cGAMP binds to and activates STING in the ER, leading to a conformational change of STING protein. Subsequently, STING is transferred to the Golgi apparatus and endosomes, where it binds and polymerizes with sulfated glycosaminoglycans (sGAGs). During this process, STING recruits TANK-binding kinase 1 (TBK1) and IRF3, and IRF3 phosphorylation by TBK1 results in IRF3 dimerization and translocation to the nucleus, finally activating expression of type I interferon. The TGN-to-plasma membrane-to-endosome pathway is essential for the activation of STING and its downstream signaling cascade due to the higher binding efficiency between STING and sGAGs under low pH conditions within the endosomes [134]. This transport route involves ARF1-dependent PtdIns4P synthesis, which produces clathrin-coated vesicles by recruiting AP-1 and GGA2 to guide STING transport from the Golgi apparatus to endosomes [135]. The termination of STING signaling depends on the endosome-to-lysosomal transport pathway, where STING undergoes ubiquitination in response to cGAMP stimulation, and the ubiquitinated STING transported to endosomes will be recognized by the recruited ESCRT complex and then degraded by the endosomal transport pathway, terminating the STING-mediated signaling transduction [136].

## Endosomal trafficking disruption is intricately linked with NDDs

The balance of sorting, degradation, and secretion of membrane proteins closely related to endosomal trafficking, allows these proteins to maintain homeostasis and adapt to changes in physiological need [137]. Increasing evidence has implicated disruption of this equilibrium in a growing number of diseases, particularly morphological and functional abnormalities of endosomes and a dysfunctional membrane vesicle transport system in NDDs. This suggests a pivotal role for the endosomal transport system in the occurrence and development of NDDs [128, 138–141].

NDDs refer to a group of neurological disorders characterized by chronic and progressive loss of neurons in either the peripheral or the central nervous system, mainly including AD, PD, Huntington's disease (HD), and amyotrophic lateral sclerosis (ALS). The pathogenesis of NDDs is intricate, yet there are common pathological features such as pathological protein aggregation, inflammation, synaptic and neuronal network detects, neuronal cell death, DNA and RNA defects, altered energy homeostasis, aberrant proteostasis, and cytoskeletal abnormalities [137]. Recent studies have shown that the regular exchange of membrane proteins mediated by endosomal trafficking is essential for maintaining synaptic plasticity. A thorough investigation into their mutual regulatory relationships will provide new strategies for the prevention and treatment of NDDs [138].

### Linking pathological protein aggregation to endosomal trafficking disorders

**Fig. 4:**
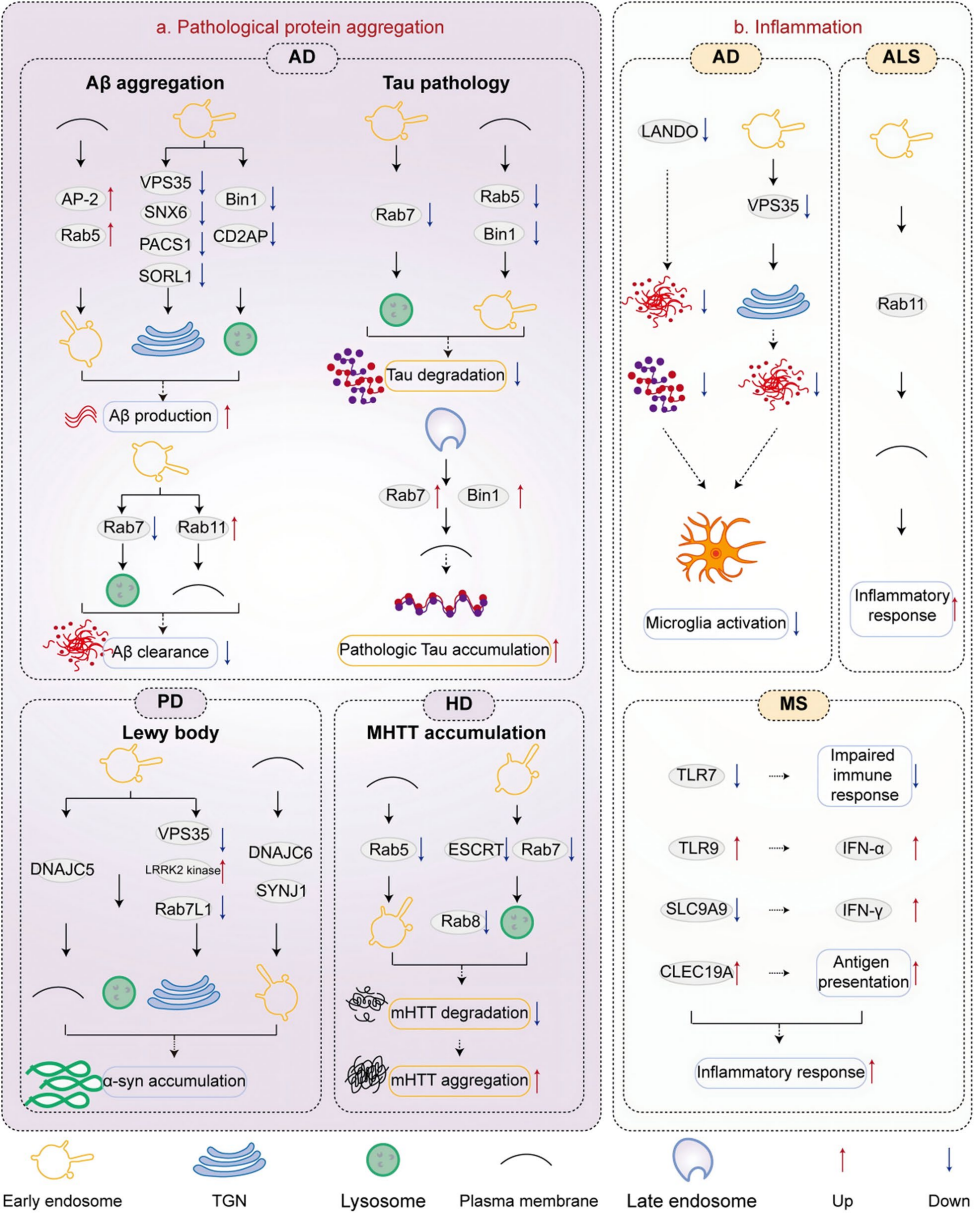
Endosomal transport disorders are implicated in pathological protein aggregation and inflammatory response in NDDs. Diverse pathways of endosomal transport modulate Aβ and Tau pathology in AD, α-synuclein aggregation in PD, and mHTT aggregation in HD (**a**). Furthermore, impaired endosomal transport can exacerbate neuroinflammation in AD, MS, and ALS (**b**)

Aberrant protein aggregation is a crucial pathological hallmark for diagnosis and classification of NDDs, such as accumulation of β-amyloid (Aβ) and hyperphosphorylated Tau (p-Tau) in AD, aggregation of α-synuclein (α-syn) in PD, and deposition of mutant huntingtin (mHTT) in HD [142–145]. Endosomal transport routes play a crucial role in the pathogenesis of NDDs by modulating the generation and clearance of pathological proteins (Fig. 4).

#### Aβ aggregation and endosomal trafficking disorders in AD

AD is characterized by neuropsychiatric symptoms including progressive cognitive impairment and memory decline. One of its pathological features involves the formation of extracellular senile plaques due to Aβ deposition [142]. The endosomes serve as the main site for Aβ production [146]. Specifically, amyloid precursor proteins (APPs) can be internalized into early endosomes, which contain β-site amyloid precursor protein-cleaving enzyme-1 (BACE1) and provide an optimal pH for APP cleavage [147]. BACE1 cleaves APP, followed by γ-secretase cleavage to release Aβ [148]. Aβ is then carried either to the lysosomes for degradation or to the extracellular space or cell surface [149]. Aβ that escapes degradation can be expelled into the cerebrospinal fluid or eliminated into the lymphatic or vascular circulation [150]. An imbalance between Aβ production and clearance leads to excessive deposition of Aβ plaques in the brains of AD patients. Failure of these redundant turnover mechanisms leads to the accumulation and aggregation of Aβ.

In the aforementioned process, mature APP and BACE1 are both carried from the Golgi apparatus to the plasma membrane, followed by internalization into the endosomes. Therefore, any disruption of the TGN-to-plasma membrane-to-endosome route may hinder Aβ generation. Overactivation of AP-2 and Rab5, which are crucial for plasma membrane internalization into endosomes, can enhance Aβ1–40 and Aβ1–42 release by upregulating BACE1 and β-CTF levels within endosomes [151, 152]. Interestingly, inhibition of Rab5 is also associated with increased production of Aβ [153].

Moreover, APP and BACE1 in early endosomes can be transported from endosomes to TGN via the endosome-to-TGN retrograde transport route, which can reduce Aβ production. Deficiency in VPS35/retromer has been observed in the hippocampus of AD patients, while deficiencies in the retromer components VPS35 and SNX6 can promote Aβ biogenesis by prolonging APP retention in early endosomes and facilitating retrograde transport of BACE1 from endosomes to TGN, respectively [154–156]. The retromer receptor SORL1 can bind to APP, and the formed SORL1/APP complex can be retrogradely transported to TGN by binding to the retromer complex or PACS1 to reduce Aβ production [157, 158].

Aβ is also produced via the endosome-to-lysosome transport pathway, which is regulated by Bin1 and CD2AP. When Bin1 levels are reduced, BACE1 becomes trapped in early endosomes and is unable to undergo axonal circulation, resulting in diminished degradation of BACE1 in lysosomes and augmented Aβ production [159]. Decreased CD2AP, on the other hand, prevents APP from being sorted for dendritic degradation as it gets stuck in the early endosome restriction membrane [160]. Bin1 and CD2AP separate APP and BACE1 apart in early endosomes through distinct mechanisms in axon and dendrites. Mutations in either gene will gradually lead to Aβ accumulation in neurons, thereby elevating the risk of late-onset AD [160].

The clearance of Aβ is also regulated by various routes of endosomal transport. Specifically, Aβ1–42 is initially internalized into Rab5-positive early endosomes and subsequently into Rab7-positive late endosomes before being transported to lysosomes for degradation, supporting the involvement of the endosome-to-lysosome trafficking in Aβ clearance [161]. Disruption of the endosome-to-lysosome trafficking may result in impaired Aβ clearance, leading to its aggregation, ultimately causing neurotoxicity. When internalized Aβ diverges from the endosome-to-lysosome trafficking and enters the endosome-to-plasma membrane pathway mediated by Rab11, it becomes shielded from degradation, leading to a significant acceleration of cellular Aβ accumulation [26].

#### Tau pathology and endosomal trafficking disorders in AD

Tau is a microtubule-associated protein in neurons. Hyperphosphorylation of tau leads to its detachment from microtubules and subsequent aggregation into neurofibrillary tangles. This process ultimately promotes the onset and progression of AD by disrupting the neuronal microtubule structure, impeding axonal function, hindering synaptic function, and even leading to nerve cell death [143]. Notably, endosomes are typically transported via microtubules, and p-tau that aggregates on microtubules traps APP-containing endosomes, thereby prolonging the residence of APP in endosomes and increasing the production of Aβ in axons. Aβ oligomers can enhance aberrant phosphorylation of tau, resulting in a vicious cycle and further exacerbating the progression of tau and amyloid pathology [162]. The endosome-to-lysosomal trafficking pathway plays an important role in the degradation and secretion of tau protein [163]. Rab5 and Bin1 are involved in the uptake of exogenous tau aggregates, and internalized tau aggregates enter the endosome-lysosomal pathway for degradation through the late endosomes labeled by Rab7 [164, 165]. Defects of the endosome-lysosomal pathway may impede tau degradation, leading to increased tau secretion and exacerbating the accumulation of extracellular pathological tau [166, 167].

#### Lewy bodies and endosomal trafficking disorders in PD

PD is characterized by bradykinesia, stiffness, postural instability, and resting tremors. One of the prominent pathological changes in PD is the emergence of eosinophilic inclusion bodies, i.e., Lewy bodies, within the cytoplasm of residual neurons in the substantia nigra [144]. α-Syn is a major component of Lewy bodies and can undergo intercellular transmission, thereby mediating their toxic effects [144]. Multiple endosomal trafficking regulators, such as DNAJC5 and DNAJC6, members of the DNAJC protein family, are crucial for endosomal trafficking of α-syn. Cytosolic α-syn is actively translocated and sequestered in endosomal membrane compartments in a DNAJC5-dependent manner. A palmitoylation-deficient mutation in DNAJC5 can result in decreased secretion of α-syn [80]. Mutations in DNAJC6 lead to aggregation of α-syn [168]. However, the kinase activity of LRRK2 regulates α-syn propagation [169, 170]. Furthermore, the phosphorylation of Rab GTPase by LRRK2 regulates lysosomal homeostasis and endosomal trafficking [171]. Heterozygous deletion of *SYNJ1* can also lead to α-syn accumulation and impaired autophagy [52].

Importantly, endosome-lysosomal transport disorders may lead to impaired α-syn clearance, and the aggregation of α-syn into toxic oligomers will further aggravate the lysosomal dysfunction, thus establishing a detrimental cycle. There is an ongoing debate regarding whether mutations in the retromer monomer VPS35 are associated with the accumulation of α-syn. D620N VPS35 knock-in mice do not consistently develop α-syn pathology in the brain even at an advanced age [172]. Dopaminergic neurons deficient in VPS35 or expressing the VPS35 D620N mutant have been reported with impaired retrograde transport of lysosome-associated membrane glycoprotein 2a (Lamp2a) from endosomes to the Golgi apparatus and accelerated Lamp2a degradation, resulting in α-syn accumulation and aggregation in the substantia nigra [173]. In contrast, a recent study found that VPS35 and α-syn do not interact to modulate neurodegeneration in multiple rodent models [172, 174]. Further investigation is required to elucidate the relationship between VPS35 and α-syn.

#### mHTT accumulation and endosomal trafficking disorders in HD

HD is an NDD caused by mutations in the huntingtin (*HTT*) gene that cause abnormal expansion of CAG repeats [175], with clinical manifestations including chorealike involuntary movements, neuropsychological symptoms, and progressive cognitive decline [176]. One of the pathological features of HD is the accumulation of mHTT [176]. HTT can be recruited to early endosomes by Rab5 through the bridging of HTT-associated protein 40 (HAP40) [141], for further degradation via the endosome-lysosomal pathway. Therefore, Rab5 inhibition leads to a reduction in mHTT entry into the endosome-to-lysosomal pathway and an increase in mHTT accumulation [177]. In addition, the ESCRT complex, Rab7 and Rab8, which play an important role in the endosome-to-lysosomal transport route, can directly participate in the degradation of mHTT, thereby protecting cells from mHTT-induced damage [178–180].

In summary, defects in the endosome-to-lysosome transport pathway in NDDs can lead to accumulation of pathological proteins or impaired autophagic clearance. Simultaneously, the augmented endosome-to-plasma membrane transport route also leads to the escape of pathological proteins from clearance, thereby increasing their aggregation. In addition, blockage of the TGN-to-plasma membrane-to-endosomal and the endosome-to-TGN transport routes plays an important role in the progression of AD, leading to prolonged retention of APP and BACE1 in early endosomes, thereby augmenting Aβ production [7, 160]. However, whether endosomal transport routes contribute to the generation of pathological proteins in NDDs requires further investigation.

### Linking inflammation to endosomal traffic disorders

Neuroinflammation is prevalent in NDDs. The sustained activation and proliferation of astrocytes and microglia can lead to increased release of inflammatory factors and chemokines with potent neurotoxic effects, triggering inflammatory responses, imbalances of energy homeostasis, synaptic dysfunction, neuronal damage and death. At the same time, neuroinflammation can expedite neurodegeneration by interacting with other pathological features of NDDs, such as pathological protein aggregation [181, 182]. Existing studies have demonstrated significant roles of endosomal transport pathways and their regulators in neuroinflammation in AD, multiple sclerosis (MS), and ALS (Fig. 4).

#### Neuroinflammation and endosomal trafficking disorders in AD

Aβ and p-Tau can stimulate microglia and astrocytes to release inflammatory factors and chemokines, thereby exacerbating the pathological progression of AD [182, 183]. The autophagy-associated protein LC3 is recruited into Aβ-containing endosomes within microglia. LC3-associated endocytosis (LANDO) in microglia serves as a pivotal regulator of microglial activation in mouse models of AD, which plays a crucial role in modulating immune activation within the central nervous system and preventing neurodegeneration [184]. Deficiency in LANDO leads to elevated levels of pro-inflammatory cytokines and Aβ protein levels, resulting in reactive microgliosis and tau hyperphosphorylation, accelerated neurodegeneration, and memory deficits [184]. Furthermore, VPS35 deficiency in microglia impairs the recycling of TREM2, which in turn affects Aβ uptake, hippocampal microglial activation, cerebral dystrophic neurite hypertrophy, and reactive astrogliosis [185]. In addition, VPS35 deficiency impairs adult hippocampal neurogenesis as well as learning and memory functions [186, 187].

Inflammatory cytokines secreted by microglia can also impair endosomal trafficking processes in other nerve cells. Brain-derived neurotrophic factor (BDNF) supports neuronal function and synaptic plasticity. Endosomal trafficking encompassing endocytosis, sorting, and transportation, is key to BDNF signaling [188]. However, the pro-inflammatory cytokine IL-1β secreted by microglia can hinder BDNF signaling by attenuating endosomal transport in hippocampal neurons [189].

#### Inflammation and endosomal trafficking disorders in MS

The inflammatory response in MS is predominantly related to endosomal TLRs. The expression and activity of TLR7 and TLR9, which are located on endosomes, are altered in MS. TLR7 activation induces B cell maturation and differentiation into plasma cells that secrete immunoglobulins (Igs), including IgM and IgG. Deficiency of TLR7 has been observed in MS patients, potentially exacerbating the impaired immune response to RNA virus infection recognized by TLR7 [190]. TLR9 recognizes viral DNA during the initial stages of viral infection and is stimulated to trigger IFN-α production in herpes simplex virus-induced MS models [191]. Additionally, in human antigen-presenting cells, the MS-associated gene *CLEC16A* promotes the formation and transportation of HLA-II-positive late endosomes to the perinuclear area [91]. A genome-wide association study in IFN-β-treated MS patients showed that SLC9A9 (an Na^+^-H^+^ exchanger in endosomes) expression is diminished in MS patients who are more likely to have repalses. An in vitro study showed that *SLC9A9* knockdown in T cells leads to increased IFN-γ expression, thereby leading T cell differentiation towards a pro-inflammatory fate [192].

#### Inflammation and endosomal trafficking disorders in ALS

ALS is caused by selective degeneration of spinal and corticospinal motor neurons and manifests as progressive skeletal muscle weakness, muscle atrophy, fasciculations, bulbar paralysis, dyspnea, and limb paralysis [193]. One of the pathological features of ALS is the presence of astrocytic hyperplasia and activated microglia surrounding both upper motor neurons and lower motor neurons [194]. Research has demonstrated that the crosstalk between Rab11 signaling and the inflammatory signaling pathway (MAPK-ERK1/2 or MAPK-AKT) triggers an inflammatory response in ALS [195].

Therefore, there exists a mutual regulation between neuroinflammatory responses and the endosomal transport system. On the one hand, the plasma membrane-to-endosomal route of endocytosis and the endosome-to-lysosome route for clearance are essential for degrading toxic pathological proteins that may induce neuroinflammatory responses. Defects of the endosome-to-TGN trafficking of the phagocytic receptor also reduce the uptake of toxic pathological proteins. On the other hand, when inflammatory factors secreted by microglia are internalized by neurons during an inflammatory response, disruptions in the endocytic transport route of plasma membrane-to-endosome in neurons may impair the uptake of neurotrophic factor. It is well established that neuroinflammation plays a crucial role in the pathogenesis of NDDs [196–198]. Previously, we have discussed that endosomal trafficking can regulate immunity by mediating the activations of TLRs, NLRP3 inflammasomes, and STING signaling; however, few studies have investigated how endosomal trafficking regulates NDD pathology through these pathways. The precise relationships between endosomal trafficking, immune response, and NDDs need to be further explored. Investigating the regulatory role of the endosomal trafficking system in inflammatory responses is a potential direction for elucidating the pathogenesis of NDDs.

## Linking dysfunctional synapses and neural networks to endosomal trafficking disorders

**Fig. 5:**
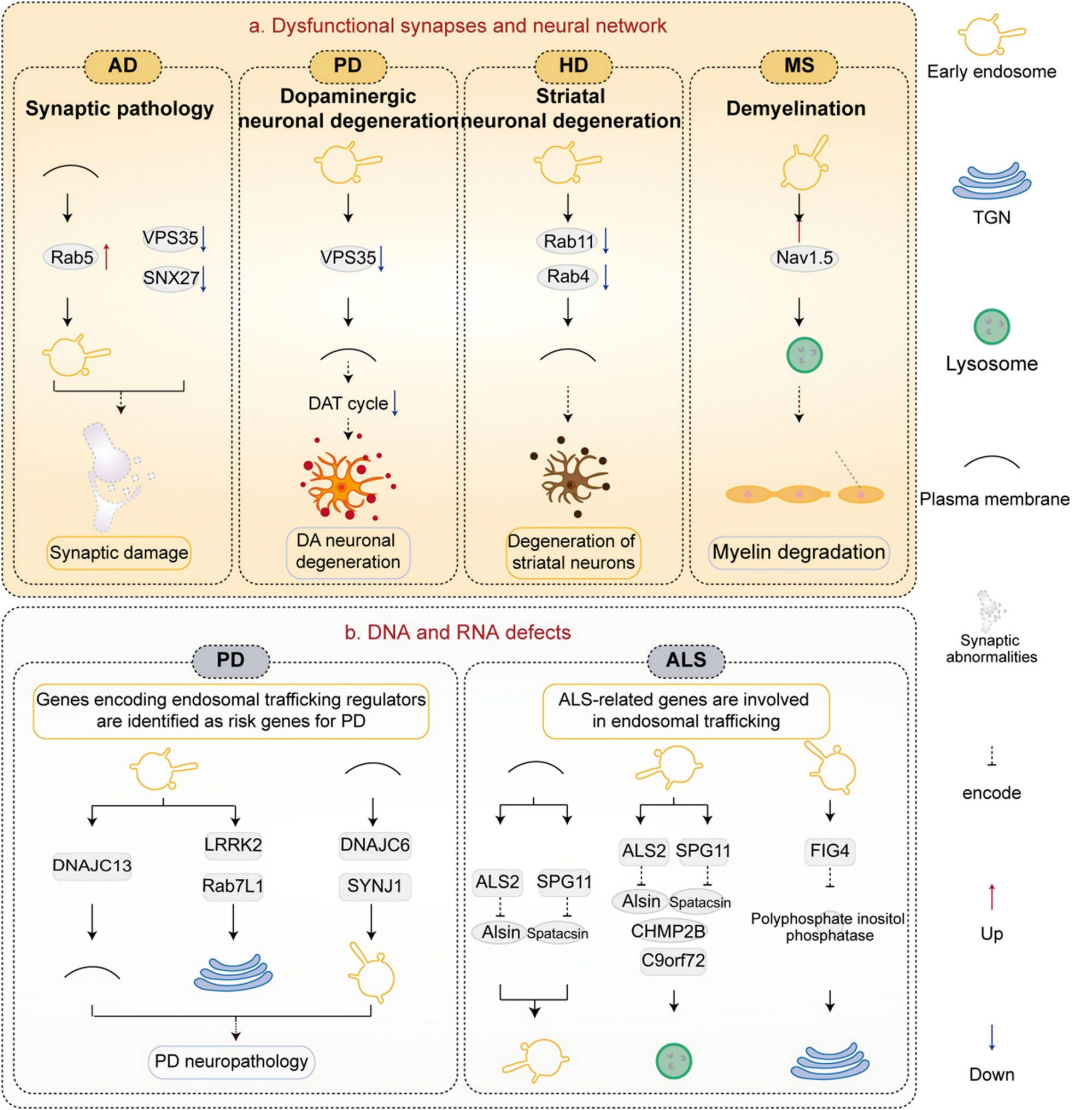
Endosomal transport disorders contribute to neuronal network dysfunction and DNA and RNA defects in NDDs. Endosomal transport disorders are implicated in synaptic and neuronal network dysfunction in NDDs including AD, PD, HD, and ALS (**a**). Additionally, multiple regulators of endosomal transport have been identified as risk genes for PD and ALS (**b**)

A synapse is a structurally defined region of a neuron where information is transmitted to another neuron or an effector cell. The intensity of synaptic transmission depends on neuronal activity. The endosomal transport system plays an important role in maintaining normal functions of the synaptic and neuronal networks. Synaptic and neuronal network dysfunction is an early event preceding neurodegeneration in NDDs (Fig. 5) [199].

### Synaptic pathology and endosomal trafficking disorders in AD

One prominent pathological feature of AD is synaptic structural abnormalities and dysfunction in affected brain regions [200]. Synaptic plasticity, including long-term potentiation (LTP) and long-term depression, represents the fundamental mechanism underlying learning and memory processes [201]. Retrosomal components that play a crucial role in endosomal sorting and transport have significant implications for axonal guidance and spine development [87, 139, 202]. Studies have shown that decreased VPS35 and SNX27 levels may impede neuronal growth and synaptogenesis, resulting in fewer excitatory synaptic connections [139, 203, 204]. Early synaptic alterations characterized by the loss of glutamate receptors can be exacerbated by depletion of VPS35 [205, 206]. VPS35 also facilitates AMPA receptor transport during NMDA receptor-dependent LTP in mature hippocampal synapses [206].

Furthermore, impairment of endocytosis from the plasma membrane to endosomes has a direct impact on axon transport. Endosomal dysfunction mediated by overactivation of Rab5 causes progressive cholinergic neurodegeneration and disrupts the hippocampal-dependent memory [207]. At the axonal terminus of normal basal forebrain cholinergic neurons (BFCNs), TrkA receptors bind to and are activated by nerve growth factor (NGF). Rab5-mediated endocytosis allows the NGF-TrkA complex to be absorbed into endosomes. Microtubules convey endosomes retrogradely to the soma, where they travel to the nucleus to carry out growth and differentiation signals. Rab5 overexpression at pathological conditions causes early endosomal expansion, disrupts NGF retrograde axonal transport, and interferes with trophic signal delivery to the soma, ultimately leading to neuronal atrophy in BFCNs [208].

### Dopaminergic neuronal degeneration and endosomal trafficking disorders in PD

A prominent pathological change in PD is the degeneration of dopaminergic neurons in the substantia nigra of midbrain. Defects in Rab7L1 (also known as Rab29) can lead to PD-related neuronal degeneration and mild cognitive impairment [209, 210]. Neuronal-specific knockout of *VPS35* results in embryonic lethality mediated by selective and robust degeneration of motor neurons in the spinal cord [210]. However, *VPS35* D620N mutation does not result in complete loss of function, as evidenced by the normal viability and longevity of homozygous D620N *VPS35*-knockin mice [172, 211]. Notably, D620N *VPS35-*knockin mice exhibit age-dependent dopaminergic neurodegeneration in the nigrostriatal pathway, and dopamine levels are significantly elevated in *VPS35* D620N transgenic mice [212, 213]. *VPS35*-D620N worms exhibit enhanced swimming-induced paralysis (a behavioral assay used to investigate the underlying mechanisms of dopamine signaling in nematodes), indicating disruption of dopamine recycling back inside neurons. In addition, VPS35-D620N mutant destabilizes the complex formed by VPS35 with Rab5, Rab11, FAM21, and dopamine transporter (DAT), thereby disrupting DAT transport from early endosomes to recycling endosomes, decreasing DAT on the cell surface, and thereby increasing dopamine in the synaptic cleft. Ultimately, dopaminergic neuronal degeneration and motor dysfunction occur [214].

### Striatal neuronal degeneration and endosomal trafficking disorders in HD

HD patients suffer severe brain atrophy in the later stages of the disease due to loss of spiny neurons in the striatum [215]. Impairment of Rab5-mediated endosomal transport of neurotrophic factors may be a pivotal event contributing to HD neurodegeneration [216]. XK is a multichannel membrane protein covalently bound to glycoprotein Kell on the surface of erythrocytes and is involved in the endocytic trafficking of manganese (Mn) [217]. Deficiency of XK on the cell surface leads to the early loss of striatal neurons. Mn, an essential trace metal highly enriched in the striatum, is deficient in HD-associated striatal neurons [218]. mHTT can impede endosome-to-plasma membrane trafficking of XK, hindering XK recycling to the cell surface and drastically decreasing the endocytic trafficking of Mn across the cell membrane [219]. Expression of Rab11 improves XK dynamics within endosomes and enhances Mn accumulation in an XK-dependent way, ultimately ameliorating HD-related degeneration of striatal neurons [217]. Furthermore, impaired Rab11 activity aggravates HD by hindering the absorption of glucose and cysteine, both vital for neuronal survival [220, 221]. Overexpression of Rab4 rescues the synaptic and behavioral dysfunction caused by the HTT-Rab4 axonal transport defects in HD [222].

### Demyelination and endosomal trafficking disorders in MS

The pathologic feature for MS-active lesions is the infiltration and engulfment of myelin fragments by macrophages. In the early stages of MS, microglia function as the primary phagocytes during demyelination and are involved in the phagocytic degradation of myelin through the endosomal-lysosomal pathway. The sodium channel Nav1.5 expressed in late endosomes and phagolysosomes within macrophages is critical for phagocytic pathway of myelin degradation in active MS lesions [223]. Furthermore, exosomes derived from endosomes participate in remyelination. Oligodendrocyte precursor cells are recruited to the lesion site and differentiate into mature oligodendrocytes, which secrete exosomes containing abundant myelinating proteins to facilitate myelin production [224].

VPS35-mediated endosome-to-TGN and endosome-to-plasma membrane trafficking play a crucial role in neuronal growth, synaptic formation, and the maintenance of glutamate receptors. In addition, loss of Rab11 may lead to defects in receptor recycling through endosomal-to-plasma membrane trafficking, as well as Rab5-mediated defects in plasma membrane-to-endosome trafficking. Both of these can impair the uptake of essential nutrients for neuronal maintenance, thereby inducing neuronal degeneration in NDDs. Consistently, Rab11 downregulation has been detected in post-mortem spinal cord specimens from ALS patients [141, 195]. The maintenance of synaptic and neuronal functions is related to various physiological processes, including glycolipid metabolism, autophagy, and immunity. Exploring whether dysfunction of endosomal transport routes affects the synaptic and neuronal networks and even neuronal cell death in NDDs represents a potential avenue for further research.

## Linking DNA and RNA defects to endosomal trafficking disorders

DNA and RNA are susceptible to spontaneous decay and damage from various intracellular or environmental factors. Persistent alterations in DNA can lead to mutations, chromosome rearrangements, DNA replication collapse, and transcriptional arrest, while RNA defects can disrupt physiological processes by disruption protein homeostasis [137]. DNA and RNA abnormalities play a key role in NDDs. A variety of genes encoding regulators of endosomal trafficking have been defined as risk factors for NDDs (Fig. 5) [225, 226].

### DNA and RNA defects and endosomal trafficking disorders in PD

In *DNAJC13* p.Asn855Ser mutation carriers, immunoreactivity of DNAJC13 was observed within Lewy body inclusions [76]. Multiple locus mutations in *DNAJC13* have been associated with an increased risk of PD [76, 227]. In addition, individuals with mutations in *DNAJC6* or *SYNJ1* develop autosomal recessive early-onset PD [228]. In a previous study, deletion of the PARK16 locus gene *RAB7L1* in primary rodent neurons, or loss of its ortholog in *Drosophila* dopaminergic neurons, resulted in degeneration similar to that observed with expression of a familial PD mutant form of LRRK2, while Rab7L1 overexpression rescued this mutant phenotype [209].

*LRRK2* mutations are common genetic causes of PD. Both Rab7L1 and LRRK2 play roles in the retrograde endosome-to-TGN trafficking. Mutations in *LRRK2* disrupt the VPS35 component of the retromer complex, resulting in lysosomal defects and impaired recruitment of lysosomal hydrolase, as well as disruptions in retromer function [209]. However, a considerable number of individuals with PD-related *LRRK2* mutations do not develop α-syn neuropathology at autopsy [229]. In addition, nearly 40% of patients with *LRRK*-PD have negative results in the α-syn seed amplification assay [230]. These findings indicate that *LRRK2* mutations can drive PD neuropathology independent of α-syn aggregation. The D620N mutation of *VPS35* is the genetic determinant of PD [231]. In addition, the R524W mutation in *VPS35* has also been identified in a case of sporadic PD and is predicted to be pathogenic [210].

### DNA and RNA defects and endosomal trafficking disorders in ALS

Recent studies have observed defects of the retromer complex in both ALS patients and rodent models [232, 233]. Numerous genes associated with familial ALS, including *FUS*, *ALS2*, *CHMP2B*, and *C9orf72*, are involved in endosomal trafficking [234–237]. *FUS* encodes a nuclear-localized RNA/DNA-binding protein. Expression of an ALS-associated mutation in *FUS* leads to the presence of large, electron-dense, filament-filled endosomes and impairs synaptic vesicle docking at the neuromuscular junction, resulting in reduced neurotransmission from motor neurons to muscles [238]. Deleterious variants of *FIG4* have been found in ALS patients. *FIG4-*encoded phosphoinositide phosphatase regulates the cellular abundance of PI(3,5)P_2_, which mediates the endosome-to-TGN retrograde trafficking [239]. The Alsin protein, encoded by the *ALS2* gene, mediates Rab5 activation and localization on early endosomes to promote endosomal trafficking. Loss of *ALS2* results in a significant decrease in endosomal motility and endosomal lysosomal transformation in neurons [240, 241]. Additionally, spatacsin encoded by *SPG11*, plays a critical role in lipid turnover by lysosomes, and mutations in *SPG11* are associated with progressive juvenile-onset ALS [242]. CHMP2B (a core component of the ESCRT machinery) participates in the endosome-to-lysosomal trafficking and is responsible for intracavicular vesicle formation within MVBs. Mutations in *CHMP2B* result in endosomal expansion and autophagosome accumulation [243]. Knockout of *C9orf72* leads to impaired endosome-to-lysosomal trafficking of STING, resulting in increased STING levels within late endosomes, decreased LC3-II-mediated lysosomal degradation, and hyperactive type I interferon responses, ultimately aggravating the inflammatory phenotype [244].

Therefore, multiple genes encoding regulatory factors for endosomal transport, such as *DNAJC13*, *DNAJC6*, and *LRRK2*, are implicated as genetic risk factors for PD. Moreover, multiple pathogenic genes of ALS can modulate various aspects of the endosomal trafficking system, providing evidence that endosomal trafficking may be involved in the occurrence and development of NDDs. Autophagy is a major biological process directly involved in protein degradation via the endosomal-to-lysosomal transport pathway. Impairment of autophagy leads to pathological features such as aberrant proteostasis and cytoskeletal abnormalities in NDDs. Furthermore, there is currently no direct evidence to demonstrate that endosomal transport disorders drive NDDs by modulating altered energy homeostasis. Nevertheless, given the pivotal role of endosomal transport pathways in glycolipid metabolism as outlined above, it is conceivable that dysfunctions of these routes may mediate alterations in energy metabolism and thereby contribute to the progression of NDDs, which is worthy of further exploration.

## Potential therapeutic agents targeting endosomal trafficking disorders

Endosomal trafficking disorders play an important role in the pathological progression of NDDs. Currently, some agents have shown potential to counteract the endosomal trafficking disorders in order to ameliorate NDD pathology, including pathological protein aggregation, synaptic and neuronal network dysfunction, inflammation, and neuronal death (Table 2).

**Table 2:** Potential medications to treat endosomal transport abnormalities in neurodegenerative diseases

Category	Drug	Disease	Preclinical models	Related mechanisms	Effect	Pathology involved	References
Activators	R55	AD	*Vps26*^–/–^ primary culture neurons	Stabilizes the core of the VPS35-VPS29-VPS26 retromer complex	Reduces endosomal release of APP and Aβ	Pathological protein aggregation	[245]
	R33	AD	Sporadic AD (SAD) and familial AD (FAD) human induced pluripotent stem cell-derived neurons	Stabilizes the retromer complex	Reduces Aβ production and tau phosphorylation	Pathological protein aggregation	[246]
	TAT-UCH-L1	AD	Primary hippocampal and cortical neurons, APP/PS1 transgenic mice treated with oligomeric Aβ	Restores endosomal conduction of BDNF/TrkB signaling	Restores enzyme activity and synaptic function	Synaptic and neuronal network dysfunction	[247, 248]
	ML-SA1	AD	Induced rat primary cortical neurons with PIKfyve inhibitor (YM201636)	Activates TRPML1	Reduces Ca^2+^ content of endolysosomes and inhibits enlargement and perinuclear aggregation of endolysosomes	Pathological protein aggregation	[140]
Inhibitors	HG-10–102-01	PD	Non-transgenic or α-synuclein transgenic mice	LRRK2-mediated phosphorylation of Rab35	Decreases levels of α-synuclein aggregates	Pathological protein aggregation	[249]
	MW150	AD	APP transgenic and tau transgenic mice	Prevents overactivation of Rab5	Improves hippocampal-dependent spatial memory deficits		[284]
	VX-745	AD	Cognitively-impaired aged rats (20–22 months)	Inhibits overactivation of Rab5	Reduces hippocampal IL-1β levels and attenuates cognitive deficits	Inflammation	[285]
	MW181	AD	Aged (~ 20 months) hTau mice	Prevents overactivation of Rab5	Significant decrease in insoluble tau aggregates greatly increases the expression of synaptophysin protein, decreases the expression of IL-1β and improves working memory	Pathological protein aggregation/ Inflammation	[286]
	NJK14047	AD	9 month 5 × FAD mice	Prevents overactivation of Rab5	Reduces Aβ deposition and degenerative neurons, improves spatial learning and memory	Pathological protein aggregation	[287]
	Bin1 mAb	AD	P301S mice expressing Tau	Lowers p-Tau level	Prolongs survival	Pathological protein aggregation	[251]
	PAO	AD	Primary cortical neurons with Aβ_1–42_ treatment	Blocks Rab5- and Rab7-mediated endocytosis	Attenuates neurotoxicity caused by Aβ_1–42_	Neuronal cell death	[253]
	CysC	ALS	N2a cells expressing SOD1	Inhibits the activity of cysteine proteases	Neuroprotection	Pathological protein aggregation	[288]
	Sodium channel blockers	MS	EAE	Inhibits the sodium channel Nav1.5 on the membranes of the endosome-lysosomal system	Protects axons	Synaptic and neuronal network dysfunction/ Inflammation	[223]

## Activators of endosomal trafficking

### Chaperones R55/R33

Pharmacological chaperones are small molecules that bind to proteins, stabilizing their three-dimensional structure and protecting them from degradation. In the VPS35-VPS29-VPS26 trimer core of retromer complex, the VPS35–VPS29 interface is the weak point in the heterotrimer structure [245]. Mecozzi et al. discovered a pharmacological chaperone R55 that specifically targets the docking site at the VPS35–VPS29 interface, making the VPS35-VPS29-VPS26 trimer core less susceptible to degradation. R55 is a derivative of thiophene-thiourea that enhances the function of the retromer complex, reduces APP in endosomes, and facilitates its removal from BACE1-dependent processing, ultimately reducing Aβ production [245]. R33, an analogue of R55, has been reported to exhibit even better efficacy in human neurons; it also facilitates the spatial separation of APP from the intracellular compartments that produce Aβ and reduces the degree of tau phosphorylation in an amyloid-independent manner [246]. Retromer pharmacological chaperones hold significant potential as a therapeutic strategy for AD and other NDDs.

### TAT-UCH-L1

The complex formed by BDNF and its receptor TrkB is retrogradely transported from axons to the cell body via endocytosis and endosomal transport, playing a crucial role in neuronal survival and plasticity. Ubiquitin C-terminal hydrolase L1 (UCH-L1) is involved in the endosomal retrograde transport of the BDNF–TrkB complex by maintaining ubiquitin homeostasis. Oligomeric Aβ disrupts the BDNF–TrkB signaling by impairing ubiquitin homeostasis [247]. Delivery of TAT-UCH-L1, a fusion protein of Uch-L1 and the HIV trans-activator protein (TAT), rescues Aβ-induced impairment of retrograde trafficking of BDNF/TrkB complex and restores hydrolase activity and synaptic function in APP/PS1 mouse models [247, 248].

### ML-SA1

Endosome-lysosomal transport disorder is an early event in the pathogenesis of AD. Transient receptor potential channel mucophospholipid 1 (TRPML1) is an important endolysosomal Ca^2+^ channel whose loss of function leads to neurodegeneration. Dysregulation of TRPML1 results in enhanced endolysosomal vacuolization and perinuclear aggregation in late-onset AD neurons [140]. Blocking TRPML1 function in primary neurons results in elevated levels of endolysosomal Ca^2+^, increased endolysosomal and perinuclear aggregation, accumulation of autophagic vesicles, and early endosomal enlargement, all of which occur in late-onset AD [140]. The TRPML1 agonist ML-SA1 has been shown to rescue these transport defects in late-onset AD neurons [140].

## Inhibitors of endosomal trafficking

### HG-10–102-01

HG-10–102-01 is a highly potent, selective, and blood–brain barrier-permeable LRRK2 inhibitor with potential for PD treatment. Bae et al. reported that HG-10–102-01 reduced Rab35 levels in α-syn transgenic mice to a level comparable to that in control mice. Additionally, it normalizes the size of enlarged endosomes and significantly reduces α-syn aggregates in the neocortex, striatum, and corpus callosum by enhancing the transport of α-syn to lysosomes [249]. Targeting LRRK2-mediated phosphorylation of Rab35 represents a promising therapeutic approach for modifying disease progression.

### MW150/Neflamapimod (VX-745)/MW181/NJK14047

Since endosomal trafficking disorders occur early in AD, therapies that normalize Rab5 activity may help slow or halt AD progression before irreversible damage occurs. The α subtype of p38 mitogen-activated protein kinase (p38α) serves as the primary regulator of Rab5 activity and its effector, and inhibition of P38α kinase activity can restore Rab5 dysregulation to reverse endosomal pathology. Brain-penetrant osmoselective p38α kinase inhibitors, including MW150, Neflamapimod (VX-745), MW181, and NJK14047, hold significant therapeutic promise for NDDs [208, 250].

### Bin1 monoclonal antibody (Bin1 mAb)

As previously mentioned, Bin1 can directly bind to tau and modulate tau pathology, thereby mediating the risk of late-onset AD. Thomas et al. have proposed that Bin1 mAb could be a potential candidate for AD treatment, as it may reduce the expression level of Bin1 [251]. Both cellular and animal evidence supports this perspective, as administration of Bin1 mAb decreases the levels of tau and p-tau [251]. Furthermore, in vivo investigations have demonstrated that treatment with Bin1 mAb prolonged survival of tau-expressing P301S mice compared to untreated ones [251].

### Phenylarsine oxide (PAO)

PAO acts as a potent inhibitor of endocytosis by effectively suppressing the internalization of cell surface receptors [252]. Internalization of exogenous Aβ1–42 mediated by clathrin-dependent processes can lead to neuronal degeneration by activating the endosome-lysosomal pathway [253]. Inhibition of Aβ endocytosis not only reduces neurotoxicity but also prevents neuronal loss during AD. Additionally, PAO exerts its neuroprotective effects by blocking Rab5- and Rab7-mediated endocytosis, further mitigating the neurotoxicity associated with Aβ1–42 [253]. This therapeutic strategy holds promise for preventing AD-related neuronal death.

### Cystatin C (CysC)

CysC is a low-molecular-weight protein that belongs to the cystatin type 2 superfamily of evolutionarily conserved cysteine protease inhibitors. Endogenous CysC is present in the endosome-lysosomal system, where it functions as an inhibitor of cathepsin activity [254]. CysC levels are significantly lower in the cerebrospinal fluid of both AD patients and ALS patients [255, 256]. Exogenously administered CysC can be internalized into cells through endocytosis and subsequently exert neuroprotective effects by inhibiting cysteine proteases (like cathepsin B) via the endosome-lysosomal pathway [257]. Therefore, CysC is a promising therapeutic candidate for preventing neurodegeneration.

### Sodium channel blockers

Given the crucial role of sodium channel Nav1.5 on the membranes of the endosome-lysosomal system in phagocytic degradation in MS, sodium channel blockers that inhibit Nav1.5 may have neuroprotective effects. In the experimental autoimmune encephalomyelitis rodent model of MS, sodium channel blockers (such as carbamazepine, lamotrigine, and phenytoin) preserve axons and enhance clinical status [258–260]. Sodium channel blockers may provide axonal protection by obstructing Nav1.5-regulated endosomal acidification, thereby limiting the effective function of endosome-lysosomal pathway degradation.

## Conclusion and prospect

This review emphasizes the critical roles of endosomal transport routes, including the TGN-(to-plasma membrane)-to-endosome, endosome-to-lysosome, endosome-to-plasma membrane cycling, and endosome-to-TGN retrograde trafficking pathways, in nutrient uptake, protein homeostasis, autophagosome delivery, maintenance of synaptic structure and function, secretion of glial inflammatory cytokines, phagocytosis, and degradation of toxic substances. We present an overview of the fundamental characteristics of endosomal transport pathways and their association with physiological processes such as glycolipid metabolism, autophagy, and immunity. Building upon this foundation, we endeavor to explore the driving role of endosomal trafficking disorders in the onset and progression of NDDs.

While it is widely acknowledged that metabolism, autophagy, and immunity play significant roles in regulating the advancement of NDDs, there is limited evidence suggesting that these connections are potential mechanisms through which endosomal transport disorders expedite the occurrence and development of NDDs. Existing reports have only indicated that deficiencies in endosome-lysosome transport routes can aggravate the pathology of AD and PD by modulating autophagy [52, 140, 184]. Henceforth, delving into the molecular mechanisms by which endosomal transport disorders drive NDDs through regulation of these essential physiological processes will bridge the gap in our understanding. We also explore the roles of endosomal transport disorders in triggering the pathological changes in NDDs and related therapeutic interventions. Regarding blood–brain barrier disruption, our current understanding is primarily focused on the vesicle transport process within endothelial cells comprising this barrier [261, 262].

In conclusion, understanding the roles of endosomal transport routes and their regulatory factors in the pathology of NDDs and the underlying mechanisms contributing to endosomal morphology and dysfunction will pave the way for novel therapeutic strategies for NDDs including AD.

## Data Availability

Not applicable.

## Abbreviations

PM: Plasma membrane
GA: Golgi apparatus
AD: Alzheimer's disease
PD: Parkinson's disease
NDDs: Neurodegenerative diseases
ILVs: Intraluminal vesicles
MVBs: Multivesicular bodies
TGN: Trans golgi network
ARFs: ADP ribosylation factors
GGAs: ADP-ribosylation factor-binding proteins
AP: Adaptor protein
ESCRT: Endosomal sorting complex required for transport
VAMP7: Vesicle-associated membrane protein 7
VPS18: Vacuolar protein sorting 18
CHMP2B: Charged multivesicular body protein 2B
SNXs: Sorting nexins
HLA-II: Human leukocyte antigen II
PACS1: Phosphofurin acidic cluster sorting protein 1
CI-MPR: Cation-independent mannose-6-phosphate receptor
LDL: Low-density lipoprotein
PtdIns3P: Phosphatidylinositol 3-phosphate
GLUTs: Glucose transporters
Gcgr: Glucagon receptor
MDSCs: Myeloid-derived suppressor cells
PtdIns4P: Phosphatidylinositol 4-phosphate
CLEC16A: C-type lectin protein 16A
TLRs: Toll-like receptors
NLRP3: NOD-like receptor thermal protein domain associated protein 3
STING: Stimulator of interferon gene
ER: Endoplasmic reticulum
IRFs: Interferon regulatory factors
Caspase-1: Cysteinyl aspartate specific proteinase 1
TWIK2: Two-pore domain Weak Inwardly rectifying + K + channel 2
sGAGs: Sulfated glycosaminoglycans
APP: Amyloid precursor protein
BACE1: β-Site amyloid precursor protein cleaving enzyme-1
LANDO: LC3-associated endocytosis
BDNF: Brain-derived neurotrophic factor
IL-1β: Inflammatory cytokine interleukin-1β
LTP: Long-term potentiation
NGF: Nerve growth factor
DA: Dopamine
Lamp2a: Lysosome-associated membrane glycoprotein 2a
LRRK2: Leucine-rich repeat kinase 2
HD: Huntington's Disease
HTT: Huntingtin
mHTT: Mutant huntingtin
HAP40: HTT-associated protein 40
MS: Multiple sclerosis
Ig: Immunoglobulin
ALS: Amyotrophic Lateral Sclerosis
UCH-L1: Ubiquitin C-terminal hydrolase L1
TRPML1: Transient receptor potential channel mucophospholipid 1
PAO: Phenylarsine oxide
CysC: Cystatin C
